# Efficient delivery of small interfering RNAs targeting particular mRNAs into pancreatic cancer cells inhibits invasiveness and metastasis of pancreatic tumors

**DOI:** 10.18632/oncotarget.26880

**Published:** 2019-04-23

**Authors:** Keisuke Taniuchi, Toshio Yawata, Makiko Tsuboi, Tetsuya Ueba, Toshiji Saibara

**Affiliations:** ^1^ Department of Gastroenterology and Hepatology, Kochi Medical School, Kochi University, Nankoku, Kochi 783-8505, Japan; ^2^ Department of Endoscopic Diagnostics and Therapeutics, Kochi Medical School, Kochi University, Nankoku, Kochi 783-8505, Japan; ^3^ Department of Neurosurgery, Kochi Medical School, Kochi University, Nankoku, Kochi 783-8505, Japan

**Keywords:** pancreatic cancer, small interfering RNA, nanoparticles, delivery system, invasion

## Abstract

We report the use of small interfering RNAs (siRNAs) against *ARHGEF4*, *CCDC88A*, *LAMTOR2*, *mTOR*, *NUP85*, and *WASF2* and folic acid (FA)-modified polyethylene glycol (PEG)-chitosan oligosaccharide lactate (COL) nanoparticles for targeting, imaging, delivery, gene silencing, and inhibition of invasiveness and metastasis in an orthotopic xenograft model. *In vitro* assays revealed that these siRNA-FA-PEG-COL nanoparticles were specifically inserted into pancreatic cancer cells compared to immortalized normal pancreatic epithelial cells and knocked down expression of the corresponding targets in pancreatic cancer cells. Cell motility and invasion were significantly inhibited by adding target siRNA-FA-PEG-COL nanoparticles into the culture medium. *In vivo* mouse experiments confirmed that when intravenously delivered, these siRNA-FA-PEG-COL nanoparticles became incorporated into human pancreatic cancer cells in mouse pancreatic tumors. Little accumulation was seen in the normal pancreas and vital organs. All target siRNA-FA-PEG-COL nanoparticles significantly inhibited retroperitoneal invasion. The siRNA-FA-PEG-COL nanoparticles against *LAMTOR2*, *mTOR*, and *NUP85*, which strongly inhibited retroperitoneal invasion and significantly inhibited peritoneal dissemination compared to the other nanoparticles, improved prognosis of the mice. Our results imply that siRNA-FA-PEG-COL nanoparticles against these six targets could have great potential as biodegradable drug carriers. In particular, siRNA nanoparticles against *LAMTOR2*, *mTOR*, and *NUP85* may hold significant clinical promise.

## INTRODUCTION

Pancreatic ductal adenocarcinoma (PDAC) is a major cause of death from cancer, with approximately a quarter of a million people worldwide dying annually from PDAC [1]. The prognosis is poor, with 1- and 5-year survival rates of only 20% and 6%, respectively [2]. About half of patients present with metastatic or end-stage disease and 35% with localized unresectable disease. Among the 20% with potentially resectable disease, very few will be cured [3], underscoring the need for more effective systemic therapies coupled with targeted agents and strategies that can improve quality of life. The current standard of chemotherapy for newly diagnosed patients with advanced PDAC is either FOLFIRINOX (5-fluorouracil, leucovorin, irinotecan, and oxaliplatin) or gemcitabine/nab-paclitaxel regimens, but the 5-year survival rate is less than 5% [4]. The gemcitabine/nab-paclitaxel regimen often causes progressive peripheral neuropathy, limiting the duration of therapy [5]. Regarding targeted therapies, genetic analysis of PDAC has yielded insights related to altered signaling pathways [6]; however, unlike other cancers, the number of sequenced PDAC genomes is relatively modest. The era of targeted therapies has offered a new avenue to search for more potentially effective strategies.

PDAC is more likely to invade and metastasize at earlier stages compared to other cancers. At the time of diagnosis, >80% of patients with PDAC have locally advanced or metastatic disease [7]. However, the mechanism and details of the molecules involved in invasion and metastasis have not yet been clarified, which hinders the development of novel treatments for suppressing the invasion and metastasis of PDAC. We recently reported that insulin-like growth factor-2 mRNA-binding protein 3 (IGF2BP3)-bound messenger RNAs (mRNAs) are localized in cytoplasmic RNA granules that are transported to the membrane protrusions of PDAC cells by a kinesin motor, Kinesin Family Member 20A [8, 9]. Knockdown of IGF2BP3 suppresses the formation of cell protrusions in S2-013 cells, resulting in a round-to-oval morphology of these cells [8]. Furthermore, a total of 2,826 IGF2BP3-bound RNAs were identified [8]. From these RNAs, those associated with cell motility, invasion, and/or metastasis were selected by gene ontology analysis [8]. An IGF2BP3-bound mRNA, ADP-ribosylation factor 6 (*ARF6*) is subsequently translated in membrane protrusions; in turn, locally translated ARF6 protein influences formation of additional membrane protrusions and thereby increases the motility and invasiveness of PDAC cells [8]. Knockdown of IGF2BP3-bound mRNAs, including Rho guanine nucleotide exchange factor 4 (*ARHGEF4*), coiled-coil domain containing 88A (*CCDC88A*), or WAS protein family member 2 (*WASF2*), with small interfering RNAs (siRNAs) inhibits the *in vitro* motility and invasiveness of PDAC cells by decreasing cell protrusions [10–12]. Thus, inhibition of IGF2BP3-bound mRNAs associated with cell motility, invasion, and/or metastasis may be effective as targeted molecular therapy, because any such therapy would inhibit the formation of cell protrusions and consequently limit cell motility and invasion of pancreatic cancer cells. This study determined the effect of six siRNAs targeting the mRNA for *ARHGEF4*, *CCDC88A*, late endosomal/lysosomal adaptor, MAPK and MTOR activator 2 (*LAMTOR2*), mechanistic target of rapamycin kinase (*mTOR*), nucleoporin 85 (*NUP85*), and *WASF2* on *in vivo* invasiveness and metastasis.

RNA nanotechnology using synthetic siRNAs has recently emerged as a method for delivery of highly promising new classes of drugs to treat human diseases. However, siRNA is highly anionic and does not readily diffuse across membrane barriers [13]. One way to enhance the delivery of siRNA to the site of action is development of a suitable delivery platform with characteristics that enable biocompatibility, a high loading capacity, protection of siRNA during transport, and high targeting ability [14]. Also, because siRNA has no functional moiety targeted to the sites of interest and its negative charge leads to poor cellular uptake owing to the electrostatic repulsion between siRNA and the cell membrane [15], such targeted delivery systems require a ligand-receptor pair that is specifically found in cancer cells. Folic acid (FA), a synthetic oxidized form of folate, has been widely used as a ligand conjugate in various cancer targeting materials [16, 17]. We previously reported that systemically administered tumor-targeting siRNA/FA-poly(ethylene glycol)-chitosan oligosaccharide lactate (FA-PEG-COL) nanoparticles are vital for delivery of siRNA to ovarian cancer site(s) in BALB/c mice bearing ovarian cancer tumor xenografts [18]. We demonstrated the uptake of siRNA/FA-PEG-COL nanoparticles into ovarian cancer cells via receptor-mediated endocytosis [18].

The present study shows the potential utility of an siRNA delivery system with FA-PEG-COL nanoparticles conjugated to six types of siRNAs targeting the mRNA for *ARHGEF4*, *CCDC88A*, *LAMTOR2*, *mTOR*, *NUP85*, and *WASF2* as targeted PDAC gene therapy.

## RESULTS

### Physical characterization of siRNA-FA-PEG-COL nanoparticles

**Figure 1:**
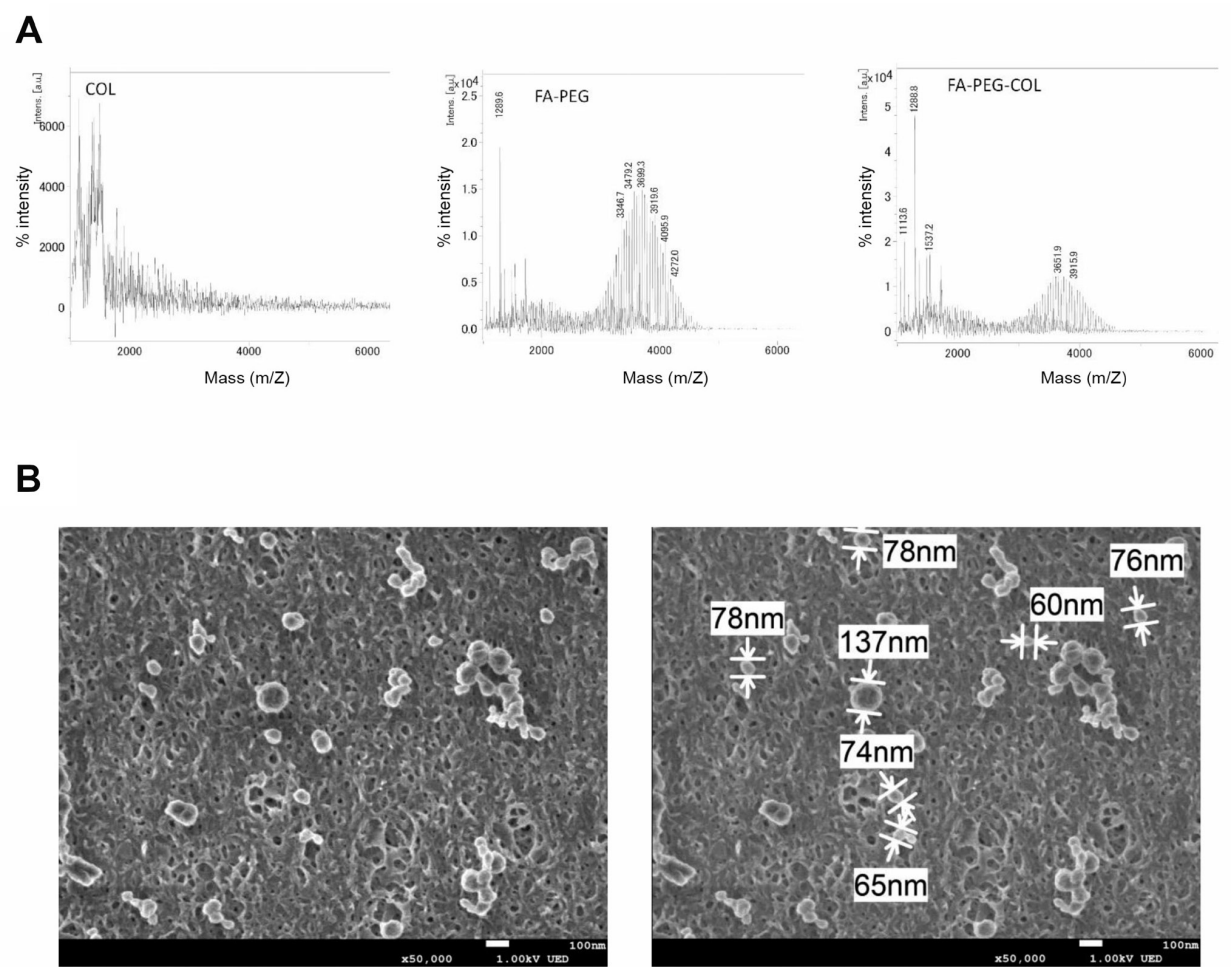
Characterization of siRNA conjugated to FA-PEG-COL. (**A**) MALDI-TOF Mass analysis of FA-PEG and FA-PEG-COL. Data are representative of three independent experiments. (**B**) SEM images of FA-PEG-COL. Scale bars, 100 nm. Data are representative of three independent experiments.

FA was linked to COL using hetero-bifunctional PEG. Matrix assisted laser desorption/ionization time-of-flight mass spectrometry (MALDI-TOF MS) was used to verify the conjugation of FA to PEG. Consistent with a previous report [18], the mass/charge (m/z) values of FA-PEG and FA-PEG-COL were 3699 and 3652, respectively (Figure 1A). The m/z value was not altered by adding COL (Figure 1A). The size of FA-PEG-COL was analyzed by scanning electron microscope (SEM). SEM images showed that the size of FA-PEG-COL was about 80 nm (Figure 1B).

### Insertion of siRNA-FA-PEG-COL nanoparticles into S2-013 and HPNE cells

**Figure 2:**
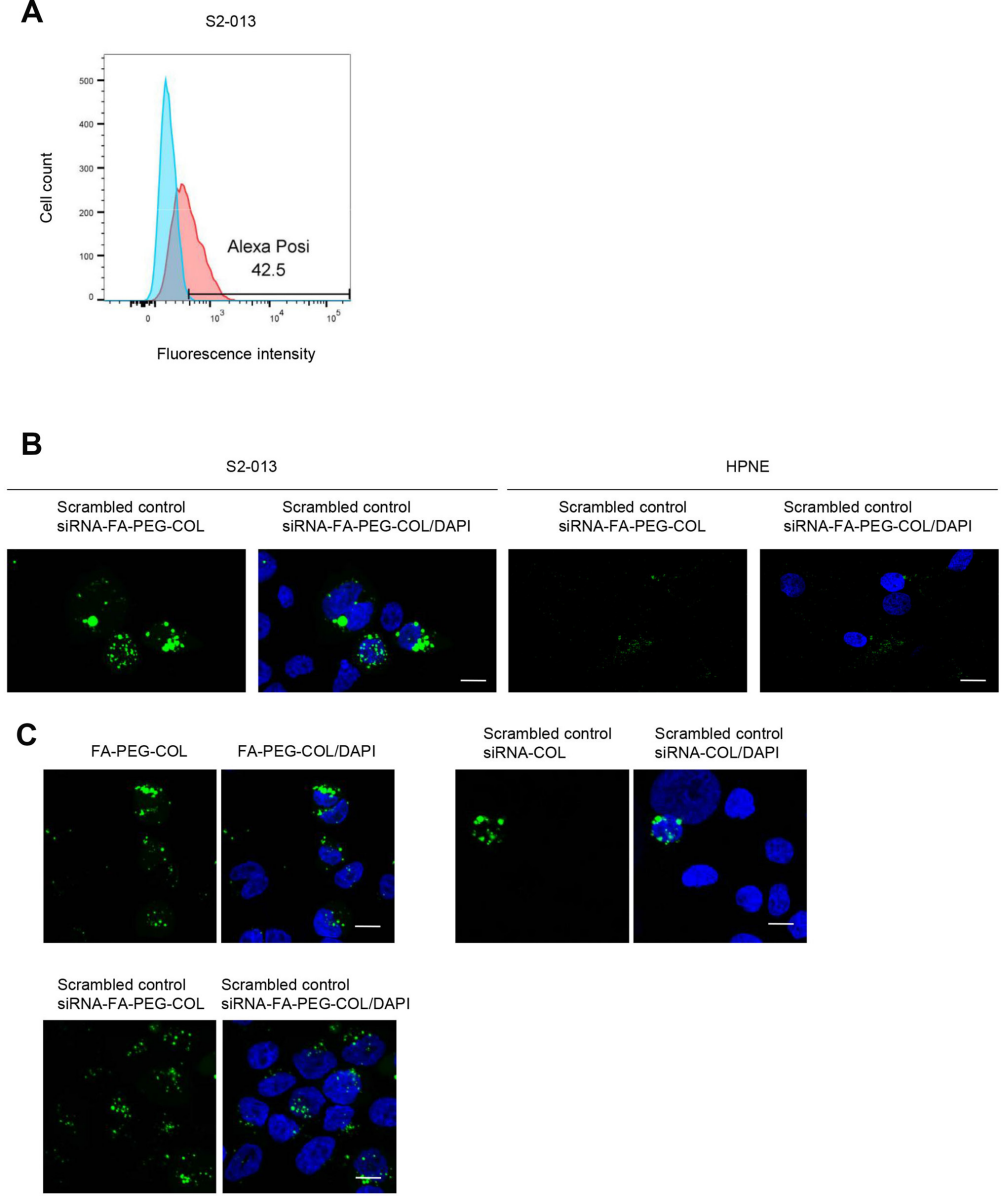
Insertion of siRNA-FA-PEG-COL nanoparticles into S2-013 and HPNE cells. (**A**) Representative flow cytometry data of Alexa 488-labeled scrambled control siRNA-FA-PEG-COL nanoparticles inserted into S2-013 cells. (**B**) Confocal immunofluorescence microscopic images of scrambled control siRNA-FA-PEG-COL nanoparticles (green) in S2-013 and HPNE cells. Blue, DAPI staining. Scale bars, 10 μm. (**C**) Confocal immunofluorescence microscopic images of FA-PEG-COL nanoparticles (green), scrambled control siRNA-COL (green), and scrambled control siRNA-FA-PEG-COL nanoparticles (green) in S2-013 cells. Blue, DAPI staining. Scale bars, 10 μm.

Alexa 488-labeled scrambled control siRNA-FA-PEG-COL nanoparticles were added to the culture media of S2-013 cells and cultured for 24 h. Flow cytometry data showed cellular uptake of scrambled control siRNA-FA-PEG-COL nanoparticles into S2-013 cells (Figure 2A). Confocal microscopy showed that abundant scrambled control siRNA-FA-PEG-COL nanoparticles were present in the cytoplasm, whereas HPNE cells displayed a weak signal (Figure 2B), strongly suggesting that the prepared siRNA-FA-PEG-COL nanoparticles were not inserted into HPNE cells.

Alexa 488-labeled FA-PEG-COL nanoparticles, Alexa 488-labeled scrambled control siRNA-COL, and Alexa 488-labeled scrambled control siRNA-FA-PEG-COL nanoparticles were cultured with S2-013 cells for 24 h, and confocal microscopy was carried out (Figure 2C). FA-PEG-COL nanoparticles and scrambled control siRNA-FA-PEG-COL nanoparticles were taken up into S2-013 cells more abundantly than scrambled control siRNA-COL, indicating that FA functioned in increasing the cellular uptake of the particles.

### Effects of siRNA-FA-PEG-COL nanoparticles on silencing of the target mRNAs in PDAC cells

**Figure 3:**
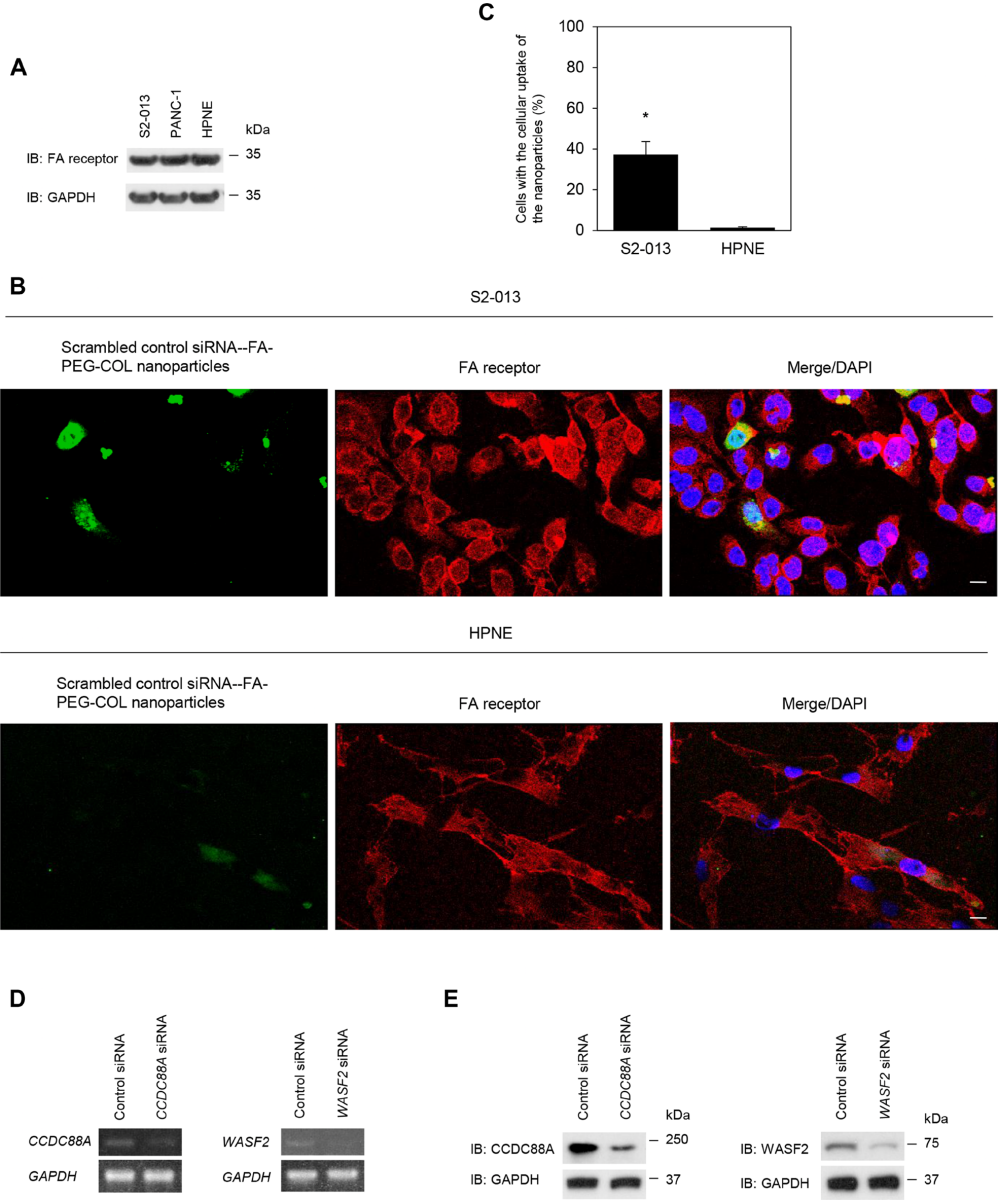
Effect of target siRNA-FA-PEG-COL nanoparticles on silencing of the targets in PDAC lines. (**A**) Western blotting was performed using anti-FA receptor antibody in S2-013, PANC-1 and HPNE cells. Data are representative of three independent experiments. (**B**) Confocal immunofluorescence microscopic images of scrambled control siRNA-FA-PEG-COL nanoparticles (green) and FA receptors (red) in S2-013 and HPNE cells. Blue, DAPI staining. Scale bars, 10 μm. (**C**) Quantification of the data shown in Figure 3B; the values represent the number of cells with the cellular uptake of scrambled control siRNA-FA-PEG-COL nanoparticles. All cells in four visual fields per group were scored. Data are derived from three independent experiments. *Columns*, mean; *bars*, standard deviation (SD). *p < 0.001 (Student’s *t*-test). (**D**, **E**) Semi-quantitative RT-PCR (D) and Western blotting (E) for CCDC88A and WASF2 in S2-013 cells incubated with scrambled control siRNA-FA-PEG-COL nanoparticles, *CCDC88A* siRNA-FA-PEG-COL nanoparticles, or *WASF2* siRNA-FA-PEG-COL nanoparticles. Data are representative of three independent experiments.

FA receptor expression was confirmed in S2-013, PANC-1, and HPNE cells by immunoblotting and immunocytochemical analyses. Immunoblotting showed almost the same level of FA receptor expression in S2-013, PANC-1, and HPNE cells (Figure 3A). To determine whether scrambled control siRNA-FA-PEG-COL nanoparticles were bound to FA receptors, immunocytochemistry was performed in S2-013 and HPNE cells (Figure 3B, 3C). Alexa 488-labeled scrambled control siRNA-FA-PEG-COL nanoparticles were added to the culture media of S2-013 and HPNE cells and cultured for 24 h. Scrambled control siRNA-FA-PEG-COL nanoparticles were taken up into the cytoplasm and FA receptors were mainly localized in the cytoplasm of S2-013 cells. A portion of the particles and FA receptors was co-localized in granules in the cytoplasm of S2-013 cells, whereas few co-localization of scrambled control siRNA-FA-PEG-COL nanoparticles and FA receptors was seen in HPNE cells. These results suggested that scrambled control siRNA-FA-PEG-COL nanoparticles bound to FA receptors were inserted into S2-013 cells.

Representative semi-quantitative reverse transcription-PCR (RT-PCR) for *CCDC88A* and *WASF2* showed that prepared *CCDC88A* siRNA-FA-PEG-COL nanoparticles and *WASF2* siRNA-FA-PEG-COL nanoparticles knocked down the expression of the corresponding mRNAs in S2-013 cells after incubation for 48 h, whereas scrambled control siRNA-FA-PEG-COL nanoparticles did not (Figure 3D). Additionally, Western blotting for CCDC88A and WASF2 showed that *CCDC88A* siRNA-FA-PEG-COL nanoparticles and *WASF2* siRNA-FA-PEG-COL nanoparticles down-regulated expression of the corresponding protein in S2-013 cells, whereas control siRNA-FA-PEG-COL nanoparticles did not (Figure 3E). Thus, *CCDC88A* siRNA-FA-PEG-COL nanoparticles and *WASF2* siRNA-FA-PEG-COL nanoparticles specifically reduced the expression of CCDC88A and WASF2, respectively, in S2-013 cells. We confirmed that other siRNA-FA-PEG-COL nanoparticles against mRNAs for *ARHGEF4*, *LAMTOR2*, *mTOR*, and *NUP85* down-regulated expression of the corresponding mRNA and protein (data not shown).

### Effects of knockdown of the target mRNAs on cell motility and invasion *in vitro*

**Figure 4:**
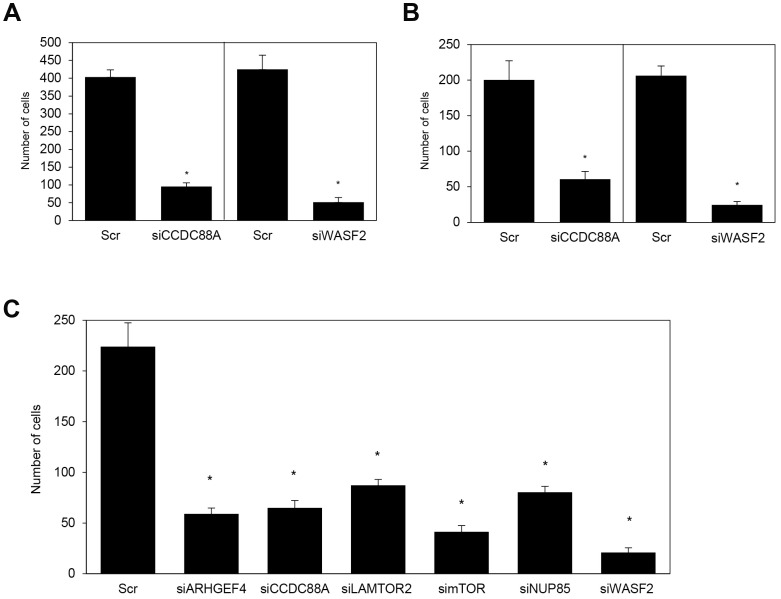
Knockdown effect of the target siRNA-FA-PEG-COL nanoparticles on cell motility and invasion *in vitro*. (**A**, **B**) S2-013 cells were incubated with scrambled control siRNA-FA-PEG-COL nanoparticles (Scr), *CCDC88A* siRNA-FA-PEG-COL nanoparticles (siCCDC88A), or *WASF2* siRNA-FA-PEG-COL nanoparticles (siWASF2). Motility (A) and two-chamber invasion (B) assays were performed. Migrating cells in four fields per group were scored. Data were derived from three independent experiments. *Columns*, mean; *bars*, SD. ^*^p < 0.05 compared to cells incubated with scrambled control siRNA-FA-PEG-COL nanoparticles (Student’s *t*-test). (**C**) S2-013 cells were incubated with scrambled control siRNA-FA-PEG-COL nanoparticles (Scr) and the target siRNA-FA-PEG-COL nanoparticles against mRNAs for *ARHGEF4* (siARHGEF4), *LAMTOR2* (siLAMTOR2), *mTOR* (simTOR), and *NUP85* (siNUP85). Two-chamber invasion assay was performed. Migrating cells in four fields per group were scored. Data were derived from three independent experiments. *Columns*, mean; *bars*, standard deviation (SD). ^*^p < 0.05 compared to cells incubated with scrambled control siRNA-FA-PEG-COL nanoparticles (Student’s *t*-test).

We previously reported that ARHGEF4, CCDC88A, and WASF2 are not associated with cell growth in PDAC cells [10–12]. We confirmed that suppression of LAMTOR2, mTOR, and NUP85 using commercial siRNA oligos also did not affect cell growth in an *in vitro* MTT assay in S2-013 and PANC-1 cells (data not shown). To determine whether knockdown of the target mRNAs using siRNA-FA-PEG-COL nanoparticles affected the motility and invasiveness of PDAC cells, *in vitro* motility and invasion assays were performed. Suppression of CCDC88A and WASF2 by adding the corresponding siRNA-FA-PEG-COL nanoparticles to the culture medium of S2-013 cells significantly inhibited cell motility in motility assays, compared to S2-013 cells incubated with scrambled control siRNA-FA-PEG-COL nanoparticles (Figure 4A). In two-chamber invasion assays, the corresponding target siRNA-FA-PEG-COL nanoparticles significantly inhibited cell invasion compared to scrambled control siRNA-FA-PEG-COL nanoparticles in S2-013 cells (Figure 4B). We confirmed that other siRNA-FA-PEG-COL nanoparticles against mRNAs for *ARHGEF4*, *LAMTOR2*, *mTOR*, and *NUP85* significantly inhibited cell invasion compared to scrambled control siRNA-FA-PEG-COL nanoparticles in S2-013 cells (Figure 4C).

### Delivery of siRNA-FA-PEG-COL nanoparticles to PDAC cells in PDAC tumors of an orthotopic mouse model

**Figure 5:**
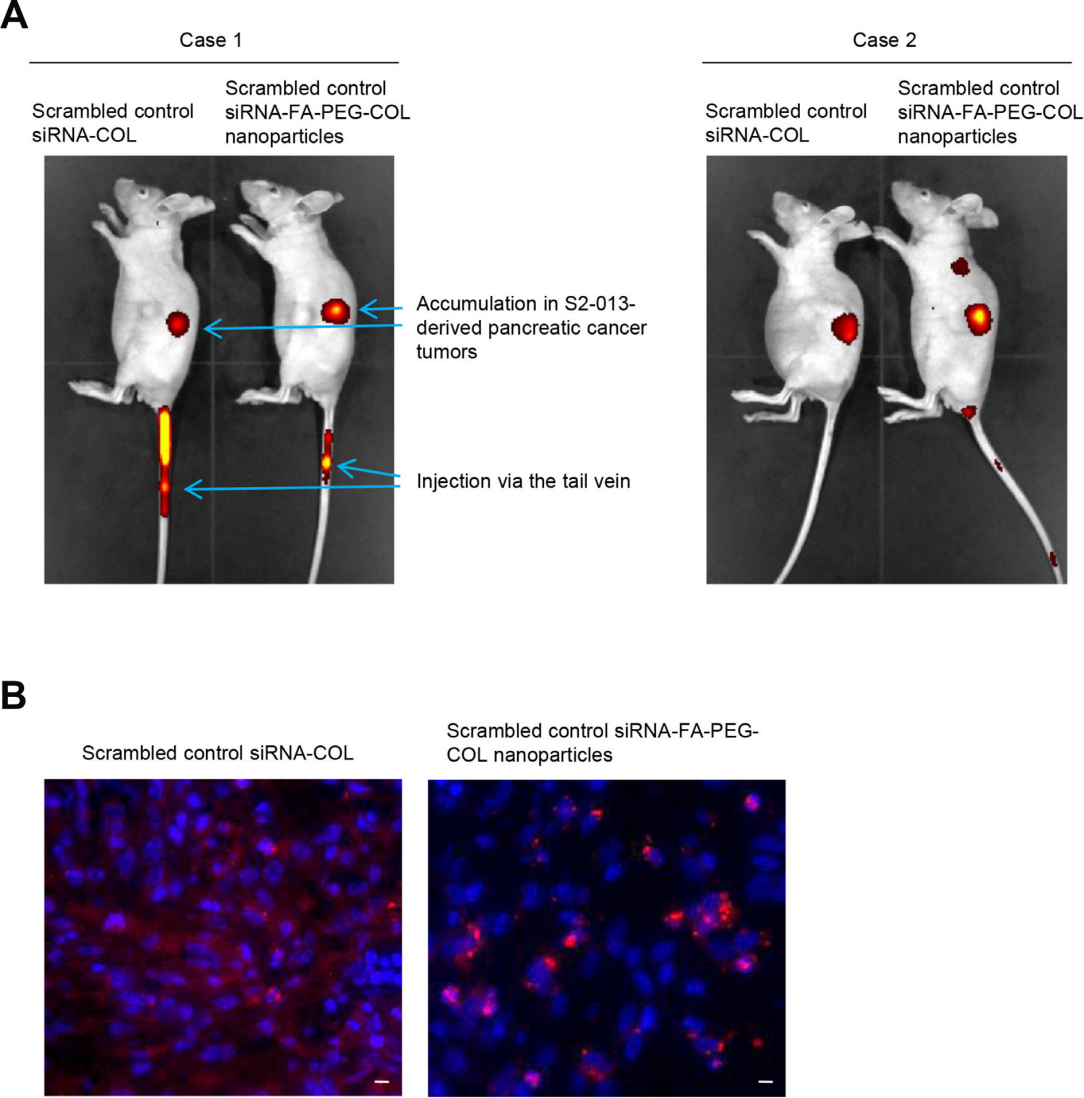
Delivery of siRNA-FA-PEG-COL nanoparticles to PDAC cells in the orthotopic mouse model of PDAC. (**A**) Whole-body *in vivo* imaging of the S2-013 tumor-bearing mice after intravenous injection of Alexa 647-labeled scrambled control siRNA-COL and Alexa 647-labeled scrambled control siRNA-FA-PEG-COL nanoparticles via the tail vein. Fluorescence intensity of the Alexa 647-labeled nanoparticles, which accumulated in S2-013-derived PDAC tumors, was measured 24 h after intravenous injection into the mice. (**B**) The S2-013 tumor-bearing mice were fixed by perfusion 24 h after intravenous injection of Alexa 647-labeled scrambled control siRNA-COL and Alexa 647-labeled scrambled control siRNA-FA-PEG-COL nanoparticles via the tail vein. Representative confocal immunofluorescence microscopic images of frozen sections of S2-013-derived PDAC tumor tissues from mice showing scrambled control siRNA-COL (red) and the siRNA-FA-PEG-COL nanoparticles (red). Blue, DAPI staining. Scale bars, 10 μm.

To determine whether siRNA-FA-PEG-COL nanoparticles were delivered to PDAC tissues, we generated an orthotopic mouse model of PDAC by surgical implantation of human S2-013 cells into the pancreas of nude mice [19]. Alexa 647-labeled scrambled control siRNA-COL and Alexa 647-labeled scrambled control siRNA-FA-PEG-COL nanoparticles were intravenously injected into nude mice once per week for six weeks after injection of S2-013 cells into the pancreas. Twenty-four hours after the last injection of the nanoparticles, *in vivo* imaging studies were performed (Figure 5A). Scrambled control siRNA-COL and scrambled control siRNA-FA-PEG-COL nanoparticles were taken up mainly into the PDAC tumors (Figure 5A). Of note, significant uptake of scrambled control siRNA-FA-PEG-COL nanoparticles into the PDAC tumors was seen compared with accumulation in the PDAC tumors of mice given scrambled control siRNA-COL (Figure 5A).

To confirm the delivery of the siRNA-FA-PEG-COL nanoparticles to PDAC cells within PDAC tumors, Alexa 594-labeled scrambled control siRNA-COL and Alexa 594-labeled scrambled control siRNA-FA-PEG-COL nanoparticles were injected intravenously into the mice administered S2-013 cells 6 weeks before. The mice were perfused 24 h after intravenous injection of the nanoparticles, and frozen sections of the S2-013-derived PDAC tumor tissues that had formed in the mouse pancreas were prepared. Scrambled control siRNA-COL were present in the tumor stroma, and uptake of the nanoparticles into PDAC cells was limited (Figure 5B). In contrast, accumulation of scrambled control siRNA-FA-PEG-COL nanoparticles into the S2-013-derived PDAC cells was markedly higher (Figure 5B).

### Effects of siRNA-FA-PEG-COL nanoparticles on silencing of the target mRNAs in the orthotopic mouse model of PDAC

**Figure 6:**
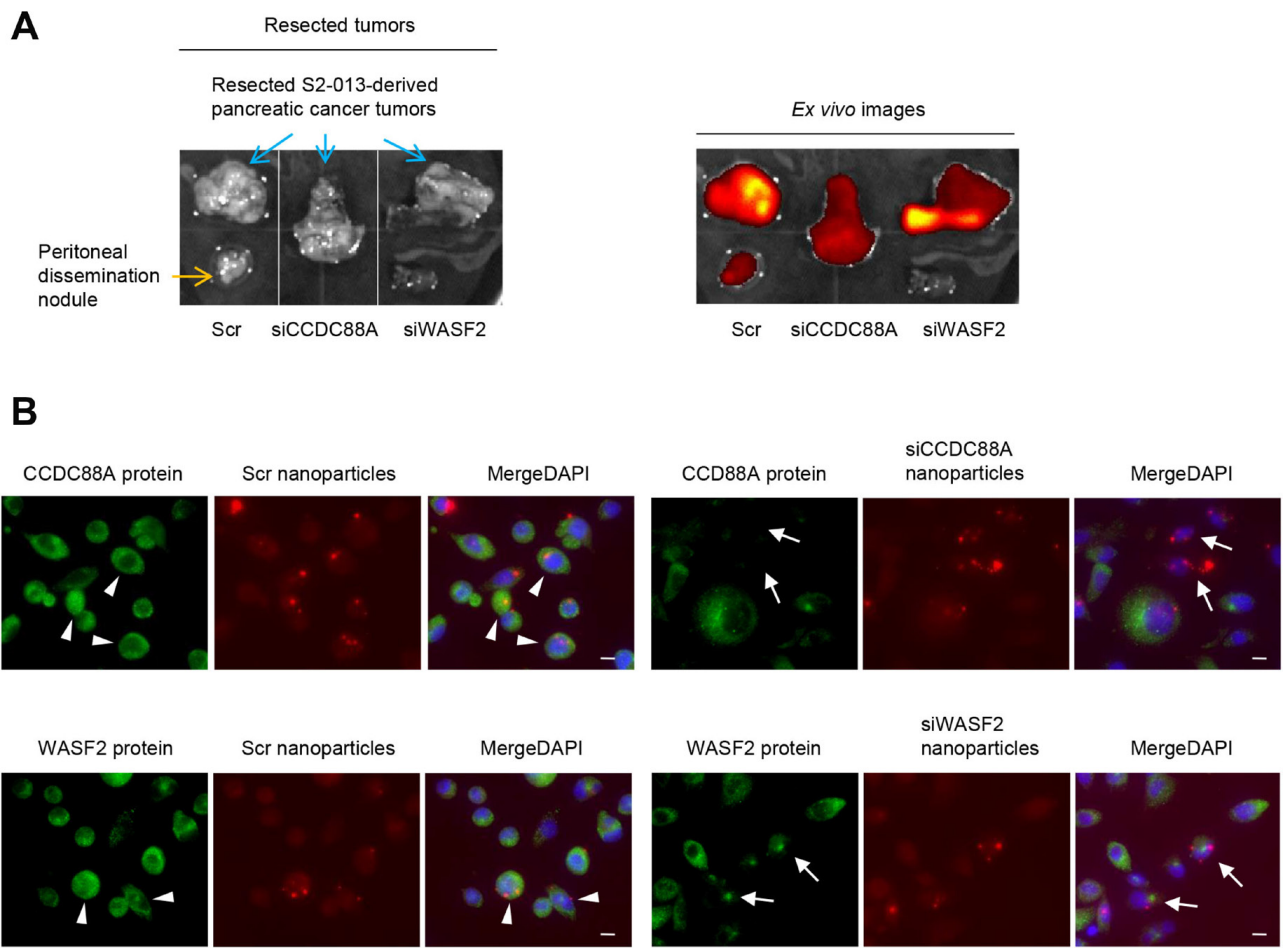
Effect of target siRNA-FA-PEG-COL nanoparticles on silencing of the targets in the orthotopic mouse model of PDAC. (**A**) *Ex vivo* images of the PDAC tumors excised from the S2-013 tumor-bearing mice after intravenous injection of Alexa 647-labeled scrambled control siRNA-FA-PEG-COL nanoparticles (Scr), Alexa 647-labeled *CCDC88A* siRNA-FA-PEG-COL nanoparticles (siCCDC88A), and Alexa 647-labeled *WASF2* siRNA-FA-PEG-COL nanoparticles (siWASF2) via the tail vein. The peritoneal dissemination nodule and mouse heart were excised from the S2-013 tumor-bearing mice after intravenous injection of Alexa 647-labeled scrambled control siRNA-FA-PEG-COL nanoparticles. Fluorescence intensity of the Alexa 647-labeled nanoparticles was measured 24 h after intravenous injection to the mouse model. (**B**) The S2-013 tumor-bearing mice were fixed by perfusion 24 h after intravenous injection of Alexa 647-labeled scrambled control siRNA-FA-PEG-COL nanoparticles (Scr) or Alexa 647-labeled target siRNA-FA-PEG-COL nanoparticles against *CCDC88A* (siCCDC88A) and *WASF2* (siWASF2) via the tail vein. Frozen sections of S2-013-derived PDAC tumor tissues were immunocytochemically stained with antibodies corresponding to the target siRNAs (green). Nanoparticles were indicated by red. Representative confocal immunofluorescence microscopic images are shown. Arrows, tumor cells showing suppression of the target proteins by the siRNA-FA-PEG-COL nanoparticles. Arrows, the target siRNA-FA-PEG-COL nanoparticle transfected tumor cells that suppress CCDC88A or WASF2. Arrowheads, the scrambled control siRNA-FA-PEG-COL nanoparticle transfected tumor cells that express CCDC88A and WASF2. Blue, DAPI staining. Scale bars, 10 μm.

To confirm the knockdown effects of the siRNA-FA-PEG-COL nanoparticles in the orthotopic mouse model of PDAC, representative experiments were carried out using siRNA-FA-PEG-COL nanoparticles against *CCDC88A* and *WASF2*. First, Alexa 647-labeled scrambled control siRNA-FA-PEG-COL nanoparticles, Alexa 647-labeled *CCDC88A* siRNA-FA-PEG-COL nanoparticles, and Alexa 647-labeled *WASF2* siRNA-FA-PEG-COL nanoparticles were intravenously injected into the nude mice 6 weeks after injection of S2-013 cells into the pancreas of the nude mice. Twenty-four hours after injection, *ex vivo* imaging studies were performed (Figure 6A). *Ex vivo* images of PDAC tumors in sacrificed mice showed the presence of scrambled control siRNA-FA-PEG-COL nanoparticles, *CCDC88A* siRNA-FA-PEG-COL nanoparticles, and *WASF2* siRNA-FA-PEG-COL nanoparticles at the 24-h time point. In addition, scrambled control siRNA-FA-PEG-COL nanoparticles were accumulated in a peritoneal dissemination nodule from PDAC tumors.

To determine the knockdown effect of the siRNA-FA-PEG-COL nanoparticles against *CCDC88A* and *WASF2*, mice that were administered S2-013 cells 6 weeks before were perfused 24 h after intravenous injection of Alexa 647-labeled siRNA-FA-PEG-COL nanoparticles against *CCDC88A* and *WASF2* or Alexa 647-labeled scrambled control siRNA-FA-PEG-COL nanoparticles. Frozen tissue sections of the human PDAC tissues derived from S2-013 cells in the mouse pancreas were prepared, and confocal immunocytochemical analysis was performed using the corresponding antibodies against each target of the siRNA-FA-PEG-COL nanoparticles. The levels of protein expression of CCDC88A and WASF2 were higher in PDAC cells that had taken up the siRNA-FA-PEG-COL nanoparticles against *CCDC88A* and *WASF2* poorly compared to PDAC cells that had efficiently taken up the nanoparticles and those that had taken up scrambled control siRNA-FA-PEG-COL nanoparticles (Figure 6B).

### Effects of knockdown of the target mRNAs on invasiveness and metastasis in the orthotopic mouse model of PDAC

**Figure 7:**
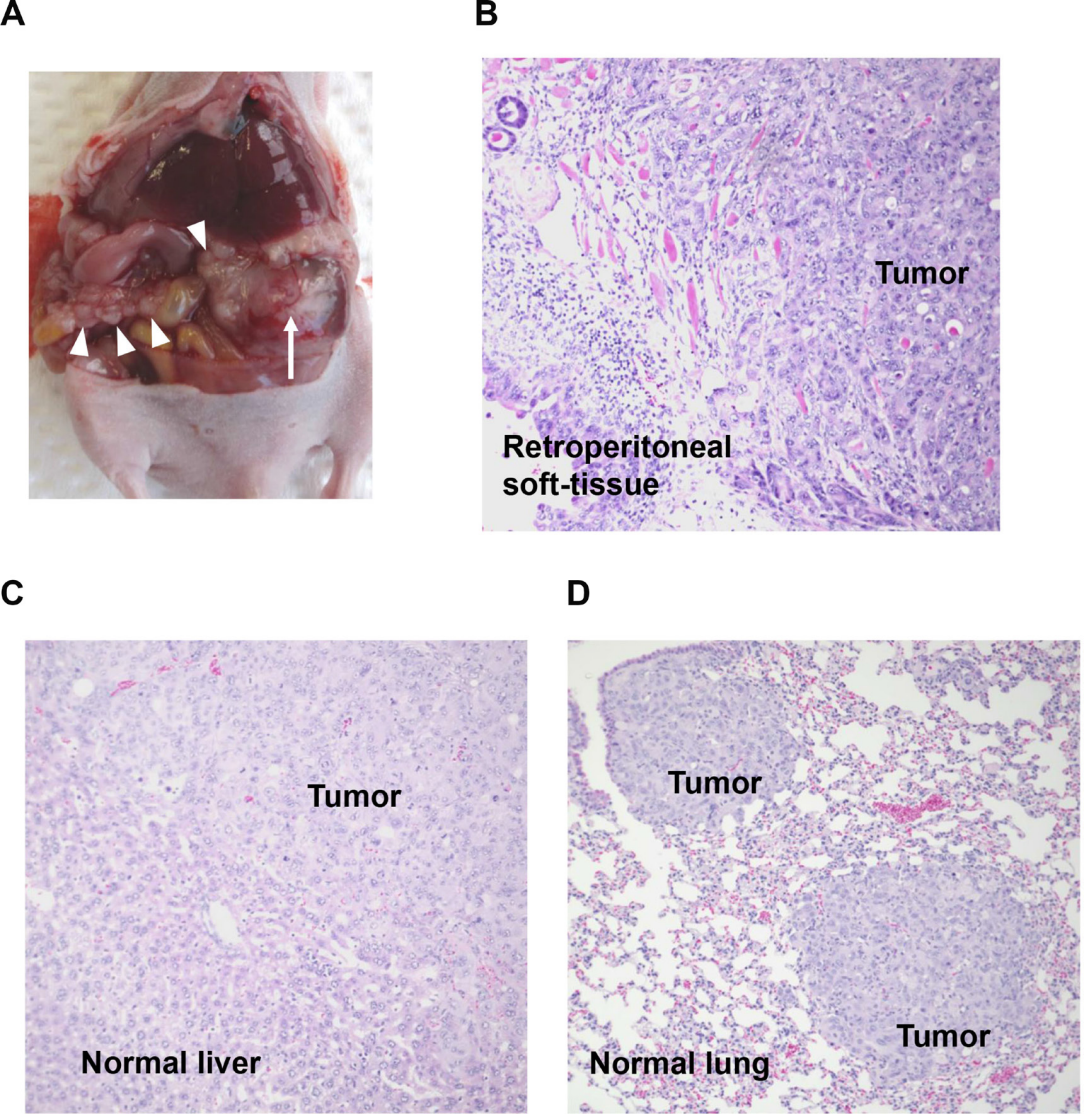
Knockdown effect of target siRNA-FA-PEG-COL nanoparticles on cell motility and invasion in the orthotopic mouse model of PDAC. (**A**) Development of carcinomatosis in S2-013 tumor-bearing mice treated with scrambled control siRNA-FA-PEG-COL nanoparticles. Arrow, primary tumor; arrowheads, dissemination nodules in the abdominal cavity. (**B**–**D**) Hematoxylin and eosin staining of representative sections of S2-013-derived PDAC tumor tissues in mice treated with scrambled control siRNA-FA-PEG-COL nanoparticles showing areas of regional invasion of the retroperitoneum (B), and distant metastases to the liver (C) and lung (D). Original magnification: × 200

To study the effects of siRNA-FA-PEG-COL nanoparticles against the target mRNAs on invasiveness and metastasis *in vivo*, the nude mouse model of PDAC established by injection of S2-013 cells into the pancreas was used. Three control groups were included: 1) scrambled control siRNA-FA-PEG-COL nanoparticles, 2) phosphate-buffered saline (PBS) alone, and 3) scrambled control siRNA-COL alone. On day 4 after injection of S2-013 cells, mice in each group received the first intravenous injection of nanoparticles or control solutions. All mice received a total of five intravenous injections once a week. Forty-two days after implantation, mice were sacrificed, sections of PDAC tissues, lung, and liver were prepared, and hematoxylin and eosin staining was performed to determine the presence or absence of peritoneal dissemination, and distant liver and lung metastases. In the control groups, 70% of the S2-013-implanted mice developed extensive peritoneal carcinomatosis (Figure 7A), and all of the mice developed hemorrhagic ascites. Invasion into adjacent organs, such as the spleen, stomach, and colon, was commonly observed in control groups. Hematoxylin and eosin staining of representative sections of S2-013-derived PDAC tumors showed adenocarcinoma with regional invasion of the retroperitoneum (Figure 7B). Histologic analysis of liver and lung metastases is shown in Figure 7C and 7D, respectively. Fisher’s exact test showed that all target siRNA-FA-PEG-COL nanoparticles significantly inhibited retroperitoneal invasion compared to the control groups (Table 1). Of note, the siRNA-FA-PEG-COL nanoparticles against mRNAs for *LAMTOR2*, *mTOR*, and *NUP85* strongly inhibited regional invasion of the retroperitoneum and significantly inhibited peritoneal dissemination. siRNA-FA-PEG-COL nanoparticles against *CCDC88A*, *ARHGEF4*, *LAMTOR2*, and *WASF2* significantly inhibited lung metastasis compared to the control groups (Table 1). The target siRNA-FA-PEG-COL nanoparticles did not inhibited liver metastasis compared to the control groups.

**Table 1:** Effect of the target siRNA nanoparticles on invasiveness and metastasis *in vivo*

	Mice (*n*)	peritoneal dissemination	Retroperitonem invasion	Liver metastasis	Lung metastasis
PBS	11	8/11	9/11	6/11	6/11
Scrambled control siRNA-COL	10	6/10	9/10	4/10	8/10
Scrambled control siRNA-FA-PEG-COL nanoparticles	9	6/9	7/9	3/9	6/9
*CCDC88A* siRNA-FA-PEG-COL nanoparticles	9	3/9	2/9^a^	1/9	1/9^a^
*ARHGEF4* siRNA-FA-PEG-COL nanoparticles	9	4/9	3/9^a^	3/9	1/9^a^
*LAMTOR2* siRNA-FA-PEG-COL nanoparticles	8	1/8^a^	1/8^a^	2/8	2/8^a^
*mTOR* siRNA-FA-PEG-COL nanoparticles	8	2/8^a^	0/8^a^	3/8	3/8
*NUP85* siRNA-FA-PEG-COL nanoparticles	9	1/9^a^	1/9a	1/9	3/9
*WASF2* siRNA-FA-PEG-COL nanoparticles	10	4/10	2/10^a^	2/10	2/10^a^

^a^*p* < 0.05 compared to controls (PBS, FA-PEG-COL nanoparticles and scrambled control siRNA)

### Disease progression and prognosis

**Figure 8:**
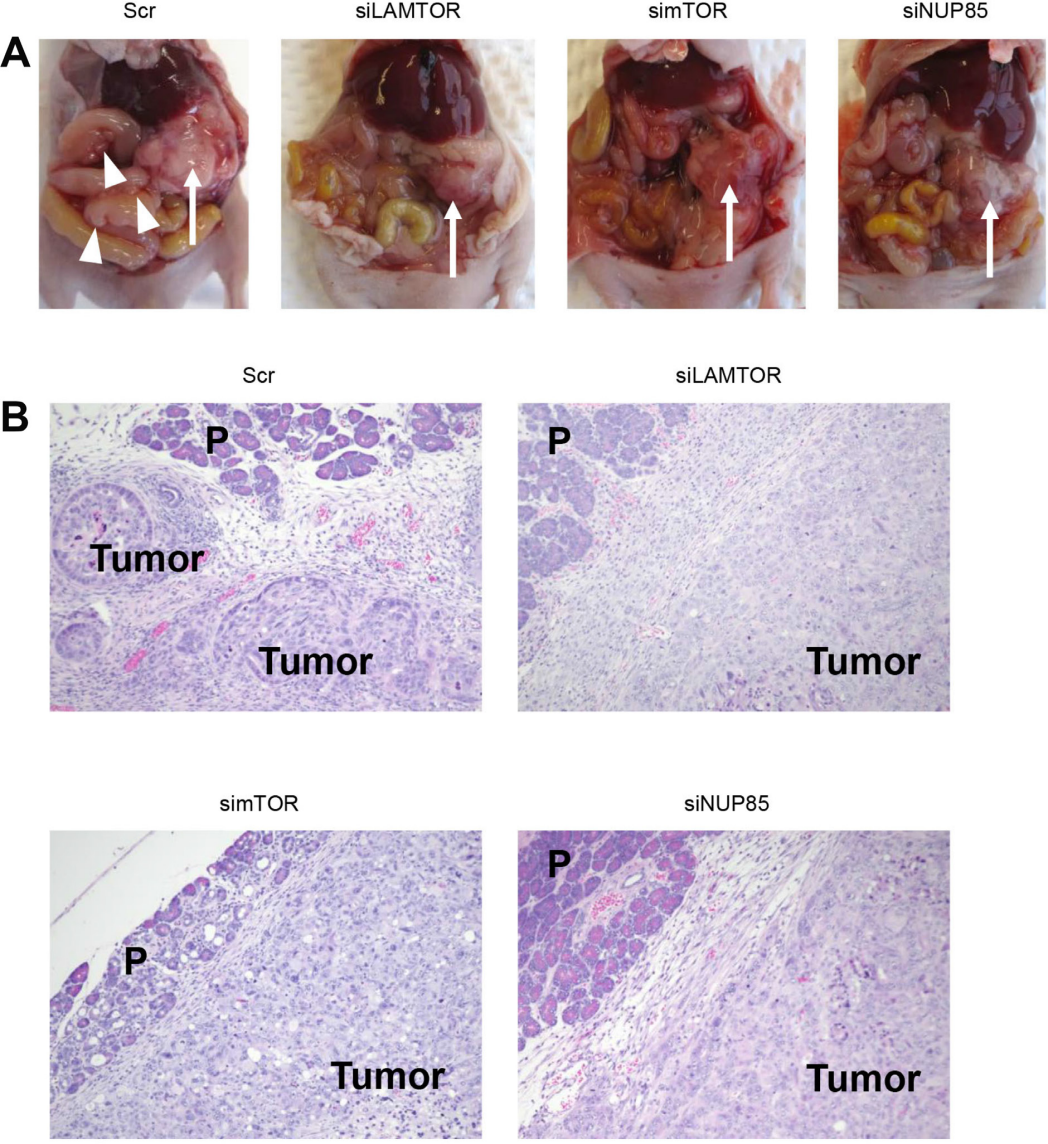
Disease progression in the orthotopic mouse model of PDAC. (**A**) Representative S2-013-derived PDAC tumor tissues in S2-013 tumor-bearing mice treated with scrambled control siRNA-FA-PEG-COL nanoparticles (Scr) and target siRNA-FA-PEG-COL nanoparticles against mRNAs for *LAMTOR2* (siLAMTOR2), *mTOR* (simTOR), and *NUP85* (siNUP85). Arrow, primary tumor; arrowheads, dissemination nodules in the abdominal cavity. (**B**) Hematoxylin and eosin staining of representative sections of S2-013-derived PDAC tumor tissues in mice treated with scrambled control siRNA-FA-PEG-COL nanoparticles or target siRNA-FA-PEG-COL nanoparticles against mRNAs for *LAMTOR2*, *mTOR*, and *NUP85*.

We observed survival of the mice until 8 weeks after implantation of S2-013 cells. Fisher’s exact test showed that mice in control groups had significantly worse survival compared to mice given the siRNA-FA-PEG-COL nanoparticles against mRNAs for *LAMTOR2*, *mTOR*, and *NUP85* (Table 2). In contrast, mice treated with the siRNA-FA-PEG-COL nanoparticles against other mRNAs including *ARHGEF4*, *CCDC88A*, *NUP85*, and *WASF2* had similar survival as control groups (Table 2). None of the surviving mice displayed extensive peritoneal carcinomatosis or hemorrhagic ascites, and the tumors in the pancreas were largely encapsulated (Figure 8A, 8B).

**Table 2:** Effect of the target siRNA nanoparticles on overall survival *in vivo*

	Total mice (n)	Alive mice at 8 week post-implantation
PBS	10	1/10
Scrambled control siRNA-COL	10	1/10
Scrambled control siRNA-FA-PEG-COL nanoparticles	10	1/10
*CCDC88A* siRNA-FA-PEG-COL nanoparticles	10	1/10
*ARHGEF4* siRNA-FA-PEG-COL nanoparticles	9	0/9
*LAMTOR2* siRNA-FA-PEG-COL nanoparticles	10	3/10^a^
*mTOR* siRNA-FA-PEG-COL nanoparticles	10	5/10^a^
*NUP85* siRNA-FA-PEG-COL nanoparticles	10	4/10^a^
*WASF2* siRNA-FA-PEG-COL nanoparticles	9	1/9

^a^*p* < 0.05 compared to controls (PBS, FA-PEG-COL nanoparticles and scrambled control siRNA)

### Toxicology study of siRNA-FA-PEG-COL nanoparticles in the orthotopic mouse model of PDAC

**Figure 9:**
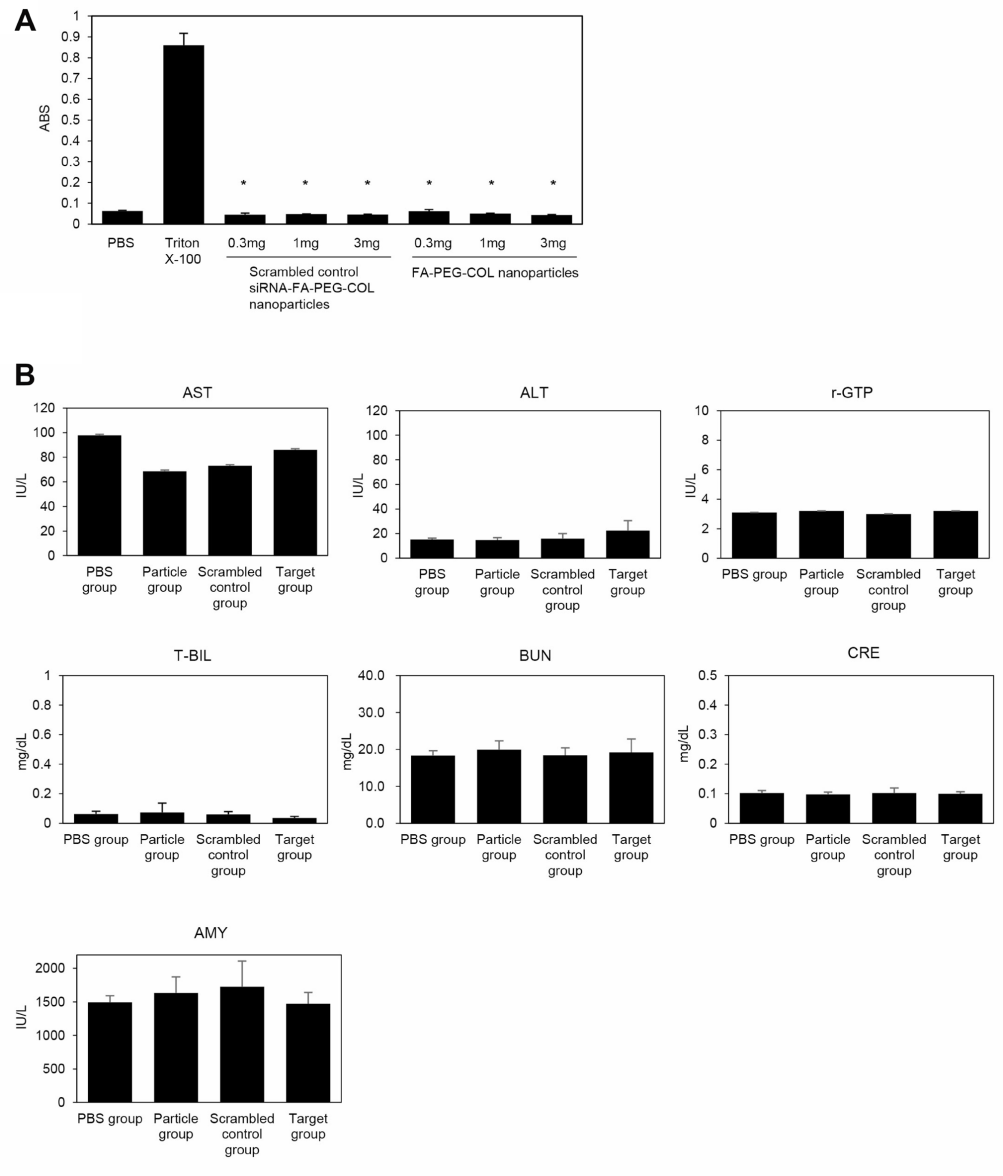
Toxicological study of siRNA-FA-PEG-COL nanoparticles. (**A**) *In vitro* hemolysis test. Diluted mouse blood was exposed to FA-PEG-COL nanoparticles and scrambled control siRNA-FA-PEG-COL nanoparticles. PBS was used as the negative control, and Triton X-100 was used as the positive control. Data were derived from three independent experiments. *Columns*, mean; *bars*, standard deviation (SD). ^*^p < 0.05 compared to the positive control (Student’s *t*-test). (**B**) Serum biochemical parameters indicating the functions of liver (ALT, AST, r-GTP, and T-BIL), kidney (BUN, CRE), and pancreas (AMY) 14 days after injection of PBS alone (PBS group), FA-PEG-COL nanoparticles alone (Particle group), scrambled control siRNA-FA-PEG-COL nanoparticles (scrambled control group), or siRNA-FA-PEG-COL nanoparticles against *CCDC88A* (target group) into mice.

The safety of siRNA-FA-PEG-COL nanoparticles was assessed. The degree of hemolysis caused by FA-PEG-COL nanoparticles and scrambled control siRNA-FA-PEG-COL nanoparticles mixed with diluted mouse blood for 1 h is shown in Figure 9A. *In vitro* hemolysis tests were negative in these two groups. The positive control chemical (Triton X-100) caused significantly greater levels of hemolysis compared to scrambled control siRNA-FA and scrambled control siRNA-FA-PEG-COL nanoparticles.

In addition to hemolysis tests, liver, kidney, and pancreas function tests were performed with blood collected after administration of siRNAs with nanoparticles (Figure 9B). Nude mice (6 weeks old; four animals per group) were given intravenous injections of PBS alone (PBS group), FA-PEG-COL nanoparticles alone (Particle group), scrambled control siRNA-FA-PEG-COL nanoparticles (Scrambled control group), or siRNA-FA-PEG-COL nanoparticles against *CCDC88A* (Target group). Finally, all mice were given a total of five intravenous injections once a week. At week 6, we collected blood from all mice to check liver [aspartate aminotransferase (AST), alanine aminotransferase (ALT), gamma-glutamyl transferase (γ-GT), and total bilirubin (T-BIL)], kidney [urea nitrogen (BUN), creatinine (CRE)], and pancreas [amylase (AMY)] functions. No group had abnormal ranges for any of the parameters compared with the PBS group. Histopathological analysis showed no abnormal pathological lesions in the lungs, livers, or kidneys of the mice (data not shown).

## DISCUSSION

PDAC is resistant to conventional chemotherapy and radiation because the cells overexpress genes such as *K-Ra*s, *p16*, *p53*, and *SMAD4* with different mutations that prevent cell death or the normal response to drugs and radiotherapy [20, 21]. The epidermal growth factor receptor (EGFR), which is upstream of K-Ras and involved in the Ras-rapidly accelerated fibrosarcoma (Raf)-MAP kinase kinase (MEK)-extracellular signal-regulated kinase (ERK) signaling pathway, plays important roles in PDAC development [22]. EGFR and its ligands are strongly upregulated in PDAC [23, 24]. The EGFR-targeted agent erlotinib, an oral EGFR tyrosine kinase inhibitor, in combination with gemcitabine has been approved, but provides only marginal benefits [25]. A clinical study (CONKO-005) indicated that adjuvant gemcitabine plus erlotinib does not improve overall survival in patients with R0 PDAC resections [26]. These previous studies indicated that inhibition of K-Ras proto-oncogene GTPase (K-Ras)-associated signaling factors may be effective as targeted molecular therapy to inhibit the growth of PDACs; however, their effects are extremely limited. Many papers assessing tumor growth of PDAC have been published, but no essential molecular targets have been identified. The major hallmark of PDAC is its early systemic dissemination and its extraordinary local tumor progression, and a standing problem in therapy for PDAC is metastatic disease [27]. Therefore, agents that target other signaling pathways associated with invasiveness and metastasis in PDAC are needed to improve outcomes of PDAC patients.

Nucleic acids such as siRNAs have tremendous versatility and target specificity [28]. siRNAs need to be modified in a manner that protects them from enzymatic degradation, thereby improving the proportion of siRNAs that reach tumor tissues after systemic administration. Subsequently, the siRNAs that reach tumor tissues need to be efficiently taken up by tumor cells. We aim to establish a delivery system with a ligand for the FA receptor that is highly expressed in the cytoplasm and membrane of PDAC cells. Intravenous injection of siRNAs against *ARHGEF4*, *CCDC88A*, *LAMTOR2*, *mTOR*, *NUP85*, and *WASF2*, which were modified with FA to allow binding to the FA receptor and PEG-COL nanoparticles, enabled target siRNA-FA-PEG-COL nanoparticle-mediated passive delivery to human PDAC tumor cells and promoted efficient siRNA endocytosis of these siRNA particles via the FA receptor on PDAC cells.

At least 22 RNA interference-based drugs have entered clinical trials, but most siRNA drug delivery systems are still in preclinical studies [29]. Targeted therapies using siRNA nanoparticles have been developed to discover better approaches for patients with other cancers. FA-conjugated BRCAA1 siRNA nanoparticles result in highly efficient siRNA delivery and reduction in the size of gastric cancer xenografts *in vivo* [30]. 7-PLK1 siRNA nanoparticles injected systemically reduce non-small cell lung cancer tumor growth in an orthotopic lung cancer mouse model [31]. Instead of focusing on the growth of PDACs, the present study showed that retroperitoneal invasion and metastasis to the liver and lung from the PDAC tumors in cancer-bearing nude mice were suppressed by administration of target siRNA-FA-PEG-COL nanoparticles against *ARHGEF4*, *CCDC88A*, *LAMTOR2*, *mTOR*, *NUP85*, and *WASF2*. The key molecules that induce PDAC tumor progression bypass one signal transduction pathway via another pathway. Thus, new drugs that target different pathways should increase the effectiveness of treatments. High expression of ARHGEF4, CCDC88A, and WASF2 predicts poor prognosis [10–12]. These proteins promote cell invasion by influencing ERK1/2 and Glycogen Synthase Kinase 3α/β (GSK-3α/β) signaling [10]; Src, ERK1/2, and AMP-activated protein kinase 1 (AMPK1) signaling [11]; and alpha-actinin 4 (ACTN4) and p27 signaling [12] in pancreatic cancer, respectively. LAMTOR2 is a member of the late endosomal/lysosomal adaptor and LAMTOR complex that regulates mTOR and ERK activation and serves as a convergence point for ERK and mTOR complex 1 (mTORC1) signaling [32]. NUP85 links activated C-C Motif Chemokine Receptor 2 (CCR2) to the phosphatidylinositol 3-kinase (PI3K)-Rac-lamellipodium protrusion cascade [33]. Inhibition of *ARHGEF4*, *CCDC88A*, *LAMTOR2*, *mTOR*, *NUP85*, and/or *WASF2* may be effective as targeted molecular therapy, because any such therapy is expected to inhibit the invasiveness and metastasis of PDACs by blocking multiple signaling pathways related to these processes. The data presented herein showed that the siRNA-FA-PEG-COL nanoparticles against *LAMTOR2*, *mTOR*, and *NUP85* strongly inhibited regional invasion of the retroperitoneum and peritoneal dissemination, and that siRNA-FA-PEG-COL nanoparticles against *CCDC88A*, *ARHGEF4*, *LAMTOR2*, and *WASF2* significantly inhibited lung metastasis.

The results from the survival study suggest that in our orthotopic mouse model of PDAC, at least partial inhibition of peritoneal dissemination and retroperitoneal invasion from PDAC tumors occurs due to administration of the siRNA-FA-PEG-COL nanoparticles against *LAMTOR2*, *mTOR*, and *NUP85*, leading to improved survival, compared with mice treated with other target siRNA-FA-PEG-COL nanoparticles. The siRNA-FA-PEG-COL nanoparticles against *CCDC88A*, *ARHGEF4* and *WASF2* significantly inhibited regional invasion of the retroperitoneum and metastasis to lung; however, they did not improve survival. This may be due to the remarkably short overall survival of our orthotopic mouse model of PDAC, and peritoneal dissemination and retroperitoneal invasion may have been a direct cause of death in our orthotopic mouse model. Free PDAC cells in the peritoneal cavity have been observed in 20-40% of human PDAC cases, even in patients without nodal involvement who underwent early resection [34]. Thus, to improve overall survival in patients with PDAC, more effective and better tolerated therapies that inhibit local and regional invasion from the tumor as well as distant metastasis are needed. The data for the siRNA-FA-PEG-COL nanoparticles against *LAMTOR2*, *mTOR*, and *NUP85* suggest that these siRNA nanoparticles could be useful for discovering better approaches for patients with PDAC.

Blood examinations from mice that were intravenously injected with siRNA-FA-PEG-COL nanoparticles showed no definite systemic dysfunction of organ systems including liver, kidney, and pancreas. No gross abnormal findings were noted in the lung, liver, kidney, or pancreas. Moreover, the result of the *in vitro* test showed that mouse erythrocytes were not lysed by FA-PEG-COL nanoparticles or scrambled control siRNA-FA-PEG-COL nanoparticles. These findings suggest that the siRNA-FA-PEG-COL nanoparticles used in this study are safe and biocompatible.

In conclusion, this study suggests the potential to develop specific siRNA-FA-PEG-COL nanoparticles targeting *LAMTOR2*, *mTOR*, and *NUP85* to inhibit the invasion and metastasis of PDAC and to improve the prognosis. These siRNA particles were delivered with high efficiency to PDAC cells, became localized in PDAC cells, and potently mediated mRNA downregulation of their targets. No definite toxic effect was noted *in vitro* or *in vivo*. This approach holds great potential for a novel therapeutic strategy for the treatment of PDACs.

## MATERIALS AND METHODS

### Antibodies

Anti-ARHGEF4 (55213-1-AP) and anti-NUP85 (19370-1-AP) antibodies were purchased from Proteintech (Chicago, IL, USA). Anti-CCDC88A antibody (MABT100) was purchased from Merck Millipore (Temecula, CA, USA). Anti-LAMTOR2 antibody (8145) was purchased from Cell Signaling (Danvers, MA, USA). Anti-WASF2 (sc-33548) and anti-mTOR (sc-1549) antibodies were purchased from Santa Cruz Biotechnology (Dallas, Texas, USA). Anti-folate receptor alpha antibody (MAB5646) was purchased from R&D SYSTEMS (Minneapolis, MN, USA).

### Cell culture

The human PDAC cell line S2-013, which is a subline of SUIT-2, the human PDAC cell line PANC-1, and HPNE immortalized normal pancreatic epithelial cells were maintained in Dulbecco’s modified Eagle’s medium (Gibco-BRL, Carlsbad, CA, USA) containing 10% fetal calf serum as published previously [19].

### Synthesis of the FA-PEG-COL conjugate

The FA-PEG-COL conjugate was synthesized as published previously [18].

### MALDI-TOF MS spectrometry of the FA-PEG-COL conjugate

Conjugation of FA to PEG was verified with MALDI-TOF Mass (Bruker autoflex Mass Spectrometer; Bruker Japan, Yokohama, Japan) by APRO Life Science Institute (Naruto, Japan).

### Fabrication of nanoparticles

Fabrication of nanoparticles was carried out as published previously [18].

### Preparation of siRNA-loaded COL and FA-PEG-COL nanoparticles

The siRNAs against *ARHGEF4* (5′-CAAGCCAG AAACCACAUUUAA-3′), *CCDC88A* (5′-AACGUUGG UUACACUACGUGA-3′), *LAMTOR2* (5′-CACCGCU GCCAUAGCCAGUAA-3′), *mTOR* (5′-ACUCGCUGA UCCAAAUGACAA-3′), *NUP85* (5′-CAGCGGCAGA UGACUGAACAA-3′), *WASF2* (5′-UAGGAUUAGAU CAUUAGCUCA-3′), and a scrambled control siRNA (5′-UUCUCCGAACGUGUCACGUAU-3′) were synthesized by GeneDesign (Osaka, Japan). siRNA (18.2 μL; 1.38 grams/μL) in RNase-free water was added to 500 μL of 0.70 mg/mL thiamine pyrophosphate solution and then added to pre-warmed COL at 62**°** C**,** or 500 μL of 6 mg/mL FA-PEG-COL solution, yielding a thiamine pyrophosphate-to-FA-PEG-COL weight ratio of 1:8.6. The nanoparticles were shaken in the dark for 30 min and incubated at ambient temperature for 30 min before use or analysis.

The morphology and particle size of FA-PEG-COL were characterized with SEM (JSM-7200F; JEOL Ltd., Tokyo, Japan) by JEOL Ltd.

### Confocal microscopy

S2-013 and HPNE cells were seeded on glass coverslips in FA-free RPMI 1640 (Sigma-Aldrich, St. Louis, MO, USA) and placed in a cell culture incubator. Cells were allowed to adhere overnight. Scrambled control siRNA was loaded with Alexa 488-conjugated FA-PEG-COL nanoparticles as described in the section called “Preparation of siRNA-loaded COL and FA-PEG-COL nanoparticles”. Alexa 488-labeled FA-PEG-COL nanoparticles and Alexa 488-labeled scrambled control siRNA-FA-PEG-COL nanoparticles were added to S2-013 and HPNE cells and cultured for 24 h at 37**°** C. The cells were then fixed with 4% paraformaldehyde, and each specimen was visualized using a Zeiss LSM 510 META microscope (Carl Zeiss, Gottingen, Germany).

### Flow cytometric analysis

S2-013 and HPNE cells (1 × 10^6^ cells) were cultured in 35-mm plates in 2 mL FA-free RPMI 1640 and placed in a cell culture incubator (95% air, 5% CO_2_ at 37**°** C). Cells were allowed to adhere overnight. Alexa 488-labeled scrambled control siRNA-loaded FA-PEG-COL nanoparticles were prepared as described in the section called “Confocal microscopy”. These nanoparticles were added to cultured S2-013 and HPNE cells for 24 h. Cells were washed three times with ice cold PBS, trypsinized, and re-suspended in PBS. Quantitative cellular uptake of nanoparticles was performed using a FACS Calibur flow cytometer (Becton-Dickinson, San Jose, CA, USA).

### *In vitro* gene knockdown efficiency

*In vitro* transfection and gene knockdown studies were performed in S2-013 cells. Cells were seeded in a 6-well plate at a density of 6 × 10^6^ cells per well in 2.0 mL FA-free RPMI 1640 containing 10% fetal calf serum and incubated for 8 h before changing to fresh FA-free RPMI 1640. A scrambled control siRNA and the siRNAs targeting mRNAs for *ARHGEF4*, *CCDC88A*, *LAMTOR2*, *mTOR*, *NUP85*, and *WASF2* were loaded into FA-PEG-COL nanoparticles and added to cultured S2-013 cells for 48 h. Gene knockdown was assessed at two levels, the protein level using SDS-PAGE followed by Western blotting and the mRNA level using semi-quantitative RT-PCR.

### Semi-quantitative RT-PCR

Total RNAs were extracted from each culture of cells using Trizol reagent (Invitrogen Life Technologies, Carlsbad, CA, USA) according to the manufacturer’s recommendations, treated with DNase I (Roche Diagnostic, Mannheim, Germany), and reverse transcribed to single-stranded cDNAs using oligodeoxythymidylic acid primers with Superscript II reverse transcriptase (Invitrogen). We prepared appropriate dilutions of each single-stranded cDNA for subsequent PCR amplification by quantitatively monitoring GAPDH as a control. All reactions involved initial denaturation at 94**°** C for 2 min followed by 21 cycles (for GAPDH) or 28 cycles (for targets) at 94**°** C for 30 sec, 58**°** C for 30 sec, and 72**°** C for 1 min on a GeneAmp PCR system 9700 (PE Applied Biosystems, Foster City, CA, USA).

### Trans-well motility assay

The trans-well motility assay using S2-013 cells was carried out as published previously [19]. A scrambled control siRNA and the siRNAs targeting mRNAs for *ARHGEF4*, *CCDC88A*, *LAMTOR2*, *mTOR*, *NUP85*, and *WASF2* were loaded into FA-PEG-COL nanoparticles and added to cultured S2-013 cells for 24 h. Cells were plated in BD BioCoat Control Culture Inserts (24-well plates, 8-μm pore size; Becton Dickinson, San Jose, CA, USA).

### Matrigel invasion assay

A scrambled control siRNA and the siRNAs targeting mRNAs for *ARHGEF4*, *CCDC88A*, *LAMTOR2*, *mTOR*, *NUP85*, and *WASF2* were loaded into FA-PEG-COL nanoparticles and added to cultured S2-013 cells for 24 h. A two-chamber invasion assay was used to assess cell invasion (24-well plates, 8-μm pore size membrane coated with a layer of Matrigel extracellular matrix proteins; Becton Dickinson) as published previously [19].

### Mice and orthotopic implantation of tumor cells

Our orthotopic mouse model of PDAC was established by surgical implantation of human PDAC cells into the pancreas of an immunocompetent host [19]. Briefly, pathogen-free female athymic nude mice (BALB/cSlc-*nu/nu*, 6 weeks of age) were purchased from Japan SLC (Shizuoka, Japan). Mice were treated in accordance with the Institutional Animal Care and Use Committee guidelines of Kochi University. S2-013 cells (8.0 × 10^5^) were surgically and orthotopically implanted into the pancreas of each mouse. Mice were treated with siRNA-loaded FA-PEG-COL nanoparticles once per week for six weeks, and then sacrificed 42 days after cell implantation. Sections of PDAC tissues, lung, and liver were prepared, and hematoxylin and eosin staining was then used to determine the presence or absence of tumor invasion into the retroperitoneum and of metastatic lesions in the lung and liver.

### *In vivo* accumulation of FA-PEG-COL nanoparticles loaded with siRNA

The siRNAs against mRNAs for *ARHGEF4*, *CCDC88A*, *LAMTOR2*, *mTOR*, *NUP85*, and *WASF2* and scrambled control siRNA-loaded FA-PEG-COL nanoparticles were prepared as described in the section called “Preparation of siRNA-loaded COL and FA-PEG-COL nanoparticles”. The siRNA-loaded nanoparticles were collected by centrifugation (13,000 × *g*, 15 min) and re-suspended in 100 μL PBS. Mice that had been surgically and orthotopically implanted with S2-013 cells into the pancreas were given via the tail vein a 100 μL bolus injection of scrambled control siRNA-COL; FA-PEG-COL nanoparticles; siRNAs against mRNAs for *ARHGEF4*, *CCDC88A*, *LAMTOR2*, *mTOR*, *NUP85*, and *WASF2*; or scrambled control siRNA-loaded FA-PEG-COL nanoparticles 4 days after implantation. Each injection of nanoparticles was given once weekly. Forty-one days after implantation, a final injection of nanoparticles labeled with Alexa 594 was given to one mouse per group, and 24 h later, fluorescence images were captured using an IVIS Spectrum *In Vivo* Imaging System (PerkinElmer, Waltham, MA, USA) with filters set at kexc 640 nm and kemi 680 nm. Each mouse was sacrificed 42 days after implantation; hematoxylin and eosin staining was then used to determine the presence or absence of tumor invasion into the retroperitoneum and of metastatic lesions in the lung and liver.

### PDAC tumor tissue perfusion

Twenty-four hours after intravenous injection of Alexa 647-labeled siRNA-FA-PEG-COL nanoparticles via the tail vein into S2-013 tumor-bearing mice, mice were anesthetized, systemically perfused with 0.9% sodium chloride followed by 10 mL of 4% paraformaldehyde, and the S2-013-derived PDAC tumors were removed. Frozen tissue sections of the S2-013-derived PDAC tumors were prepared, and each specimen was visualized using a VK-X1000 microscope (Keyence, Osaka, Japan).

### Hemolysis assay using siRNA-loaded nanoparticles

FA-PEG-COL nanoparticles and scrambled control siRNA-FA-PEG-COL nanoparticles were suspended in PBS at concentrations of 0.3, 1.0, and 3.0 mg/mL. Mouse blood was collected by cardiac puncture. Red blood cells (RBCs) were collected by centrifugation (1500 ×*g*, 10 min at 4**°** C) and diluted 100 times with pre-chilled PBS. RBCs and nanoparticles (250 μL each) were added to a 1.5-mL tube and shaken gently for 1 min. The samples were incubated at 37**°** C for 60 min. The samples were centrifuged (1500 ×*g*, 15 min at 4**°** C), and the absorbance of the supernatants was recorded at 541 nm. PBS was used as the negative control, and Triton X-100 (2% w/v) was used as the 100% lysis positive control.

### Blood test

Pathogen-free female athymic nude mice (BALB/cSlc-*nu/nu*, 8 weeks of age) were given via the tail vein a 100-μL bolus injection of FA-PEG-COL nanoparticles, siRNAs against *CCDC88A* mRNA, or scrambled control siRNA-loaded FA-PEG-COL nanoparticles. Nanoparticles were given once weekly. After 6 weeks, each mouse was anesthetized, and terminal blood collection was performed from the axillary vessels. After centrifuging at 1500 ×*g* at 4° C for 15 min, the cell-free supernatant serum was collected. AST, ALT, γ-GT, T-BIL, AMY, BUN and CRE levels were used to detect liver, kidney, and pancreas function (FUJIFILM Wako Chemicals, Osaka, Japan).

### Statistical analysis

StatFlex software (Ver6; YUMIT, Osaka, Japan) and SAS software (Ver9.1.3; SAS Institute, Cary, NC, USA) were used for statistical analysis. The significance of differences between groups was determined using the two-tailed Student’s *t*-test or Fisher’s exact test, as appropriate. Cumulative survival rates were calculated using the Kaplan-Meier method using R (version 3.3.3; The R Foundation, Wien, Austria). For all analyses, *p <* 0.05 was considered statistically significant.

## Abbreviations

PDAC: pancreatic ductal adenocarcinoma
siRNA: interfering RNA
FA: folic acid
PEG: polyethylene glycol
COL: chitosan oligosaccharide lactate
SEM: scanning electron microscope
RT-PCR: reverse transcription-PCR
EGFR: epidermal growth factor receptor
Raf: Ras-rapidly accelerated fibrosarcoma
MEK: MAP kinase kinase
ERK: MEK-extracellular signal-regulated kinase
GSK: glycogen synthase kinase
PI3K: phosphatidylinositol 3-kinase
